# Epigenetic effects of *in utero* bisphenol A administration: Diabetogenic and atherogenic changes in mice offspring

**DOI:** 10.22038/ijbms.2019.29909.7357

**Published:** 2019-05

**Authors:** Umbreen Bano, Samreen Memon, Muhammad Yaqoob Shahani, Pashmina Shaikh, Sameena Gul

**Affiliations:** 1 Department of Anatomy, Liaquat University of Medical and Health Sciences, Jamshoro, Pakistan

**Keywords:** Atherogenic, Bisphenol A, Diabetogenic, Kidneys, Pancreas, Uterus

## Abstract

**Objective(s):**

Bisphenol A (BPA) that is a monomer of plastic products may possibly interfere with epigenetics and be involved in onset and progression of several diseases. This study was aimed to detect the epigenetic effects of *in utero* BPA exposure in mice offspring.

**Materials and Methods:**

All experiments were performed according to the national guidelines for laboratory animals and after ethical approval. Thirty adult BALB/c female mice were divided into 3 equal groups, G1 (controls), G2 (ethanol 0.10 ml/100ml of PBS so that final concentration would be 0.01%) vehicle control and G3 (BPA 10 mg/kg). Chemicals were given twice a week throughout the pregnancy. Once delivered at term, female offspring were observed for body weight, behavior and movements. Blood glucose, serum insulin, cholesterol and high-density lipoprotein cholesterol (HDLc) were measured at 5 and 15 months postnatal. Animals were sacrificed at 15 months and pancreas, kidney, adipose tissue and uterine tissue were taken and stained with either Hematoxylin and eosin (H & E) or immunostaining and examined under light microscope.

**Results:**

Offspring of group G3 revealed abnormal changes of body weight, behavior and movements. Blood glucose, serum insulin, cholesterol and HDLc were high in group G3 offspring compared to controls. H & E staining showed changes in the parenchyma of pancreas, kidneys and uterus, which were confirmed by staining with anti- islet-1, kidney-specific (Ksp) cadherin, and anti- MLH antibody.

**Conclusion:**

*In utero* exposure of BPA exerts diabetogenic and atherogenic effects with less parenchymal tissue in endocrine pancreas, kidney and uterus.

## Introduction

Bisphenol A (BPA) was the first environment-related toxic substance, which was identified to cause alterations in the epigenetics within Avy mice (1). Beverage cans, thermal paper, food containers, utensils and water pipes contain BPA (2, 3). BPA, used in the “Epoxy resins”, is the source of formation of GP (gutta percha” which is frequently used in the dental therapy (4). Its hormone like properties makes its usage in foods industry questionable. BPA-made cans and milk bottles are now strictly prohibited because of potential toxic effects (5). Humans are frequently exposed to BPA as it is a component of polycarbonate plastics, resins lining food/beverage containers, and additives in a variety of consumer products. Over 6 billion pounds are produced worldwide annually, and several studies have reported that the levels of BPA in human tissues are in the parts per billion ranges (6-8). BPA impairs the normal functioning of the “Endocrine system” by both stimulating and suppressingthe mechanisticpathways of hormones action or by altering the signal transduction. It also interferes with the hormone synthesis. Disturbed maternal endocrine system indirectly affects the intrauterine development and growth of the fetus, which in turn plays a role in the onset and progression of several diseases in adulthood (9). Previous experiments on primates suggest that BPA plays role in the impairment of carbohydrates and fat metabolism by altering the insulin signaling (1, 5). The present study was conducted to detect the epigenetic effects of *in utero *BPA administration on metabolic functions and microscopic changes in different organs of mice offspring related to metabolic syndrome.

## Materials and Methods

This experimental study was conducted at the Animal House Sindh Agricultural University Tando Jamand Molecular Biology Laboratory, Medical Research Centre, Liaquat University of Medical and Health Sciences Jamshoro, Pakistan, after institutional ethical approval. 

For this purpose, 12 adult female albino mice were housed individually in polypropylene cages (dimensions: length, 39 cm; width, 28 cm; and depth, 14 cm) with rice husk bedding (autoclaved) of 20 mm thickness. Animal house was well ventilated with standard lighting of 12 hr light–dark cycle. Temperature and relative humidity were maintained at 22±2 ^°^C and 55±10%, respectively.

***Experimental protocol***

Mice in early estrus were bred overnight and mating was confirmed by sperm positive smears, denoted as day 0 of pregnancy. These female mice were grouped as; Group G1(conrol) phosphate-buffered saline (PBS), Group G2 (Ethanol 0.01%) and Group G3 (BPA 10 mg/kg, dissolved in 0.01% ethanol). The doses of chemicals were given twice weekly as 0.1 mlsolutionthroughout pregnancy via intraperitoneal (IP) route with 1 ml disposable syringe. During this period, body weight, movement, and behavior of animals were measured on weekly basis. Once delivered at term around 21 days on average, litter size was adjusted to 10 per group, and all litters were allowed to feed *ad libitum* up to the age of 15 months. Mice were examined grossly for body weight, behavior and movement. Biochemical analysis of cholesterol, high-density lipoprotein, insulin and glucose was performed at the age of 5 and 15 months by collectingblood samples through cardiac puncture, before the sacrifice of animals. Blood was centrifuged at 4000 rpm for 15 minutes to separate sera, which were stored at -20 ^°^C. Animals were anaesthetized with ketamine and xylazine followed by cervical dislocation euthanasia. Fine dissection was carried out to dissect the pancreas, kidney, adipose tissue and uterus. Organs were preserved in 10% formalin except adipose tissue, which was preserved in neutral-buffered saline. 

***Body weight measurement***

Body weight of the offspring was measured by electronic weight machine in grams. 

***Observing behavior***

Behavior of offspring was examined according to the behavior scale comprised of five grades that include Grade 0: No observable deficit, Grade 1: slightly abnormal gait, Grade 2: markedly abnormal gait, Grade 3: significant mobility problems, Grade 4: Immobility for more than 24 hours, Grade 5: tense and nervous on handling (10). 

***Observing movement***

Movements were observed on the criteria previously described by Robert and his colleagues (11), and included 10 grades (Table 1).

***Biochemical testing***

Blood and sera were used for the estimation of blood glucose level by glucose oxidase and serum insulin ELISA assay kit on Spectrophotometer Hitachi 902 (Roche diagnostics, USA), respectively. Blood cholesterol and HDL cholesterol (HDLc) were determined by enzymatic colorimetric method using commercially available kits on Microlab Chemistry Analyzer (Micro Lab 300 Spectrophotometer,Roche USA).

***Haematoxylin and eosinstaining***

Fixed paraffin embedded tissue pieces were cut into 3-5 µm thick sections by microtome, and stained with haematoxylin &eosin (H&E) for microscopic examination (12).

***Immunohistochemistry***

For immunohistochemistry (IHC)analysis, tissue slides were dried in the oven for 25 to 30 minutes at 80 ^°^C before dewaxing with xylene, alcohol and then distilled water. Slides were washed with 3 % hydrogen peroxide (H_2_O_2_) for 10 minutes followed by thorough rinsing with distilled water to block nonspecific antibodies. For antigenretrieval, slides were then placed in to pressure cooker for approximately 8 min at 120 ^°^C with EDTA buffer and were let to cool down by keeping in the cooker for more 10 min. Remaining EDTA buffer was cleared off by rinsing slides with PBS. The primary antibodies for pancreas (Anti-islet 1 antibody) [1B1], kidney (Ksp-cadherin (kidney-specific cadherin) and uterus anti-MLH-1 that were purchased from Sigma-Aldrich UK were applied, and the slides were kept for 1 hr atroom temperature. Slides were washed three times with PBS buffer to clear the antibody. Afterwards, amplifier (link) was applied on slides for 15 min and washed with PBS buffer. Secondary antibody was applied and slides were kept for 10 to15 minu in dark at room temperature. Slides were washed with 3X PBS buffer and then with distilled water to remove secondary antibody. Once washed, 1 drop of 3,3’-Diaminobenzidine (DAB) chromogen (5:1) was applied for 5 min. Final rinse with distilled water was performed to clear off the DAB. Slides were then counter stained with haematoxylin and rinsed with distilled water for 5 to 10 min. Stained slides were then placed into oven for drying before being mounted with DPX (A mixture of distyrene (a polystyrene), a plasticizer (tricresyl phosphate), and xylene. 

***Sudan black staining***

Sudan Black staining was used for adipose tissue staining according to the protocol described in Theory and Practice of Histological Techniques (12). 

***Statistical analysis***

Data were analysed by SPSS 22.0 (IBM, Incorporation USA). Kruskall- Wallis test was used for normality of data distribution. One way analysis of variance (ANOVA)was also used to analyse the numerical data variables among groups with descriptive statistics. *Post hoc* Tukey Kramer was performed to analyze the level of statistical significance between and among groups. Categorical data was analyzed by Chi-square testing. Data variables were analyzed at 95% confidence interval (*P*-value≤ 0.05). 

## Results

***Behavior and movement***

Abnormalities of behavior and movement were revealed in G3 offspring. Experimental group G3 revealed abnormal behavior and movements of grades 3-6 (*P* value=0.0001). Offspring of G1 and G2 were moving quickly around the cage at 4month (G1 vs. G2 *P>*0.05), whilst the offspring of G3 show statistically significant behavior and movement abnormalities (*P* value=0.0001) (Tables 2, 3).

***Metabolic functions***

Body weight, blood glucose, serum insulin, serum cholesterol and HDLc were high in Group G3 (BPA), which are shown in Tables 4 and 5. 

**Figure 1:**
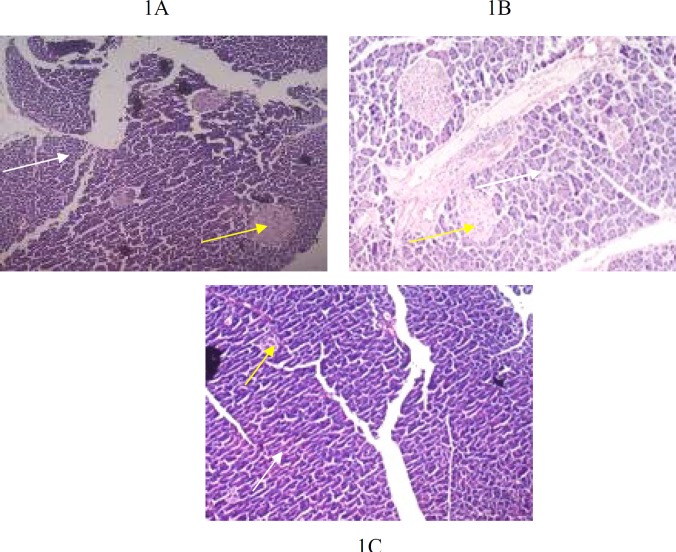
1A, 1B (G1, G2): Photomicrograph of 5 µm section of pancreas stained with H&E staining, showing normal pancreatic architecture at X20 magnification. White arrow indicates exocrine part and yellow arrow indicates Ielts of Langerhan’s

1C (G3): Photomicrograph of 5 µm section of pancreas stained with H&E staining, showing distorted pancreatic architecture at X20 magnification.white arrow indicates exocrine part and yellow arrow indicates small sized Ielts of Langerhan’s;

**Figure 2:**
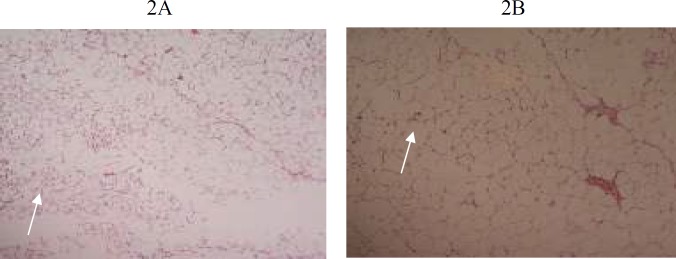
2A, 2B (G1, G3): Photomicrograph of 5 µm section of adipose tissue stained with H&E staining, showing normal adipocytes at X20 magnification. White arrow indicates adipocytes

G: Grade

**Figure 3:**
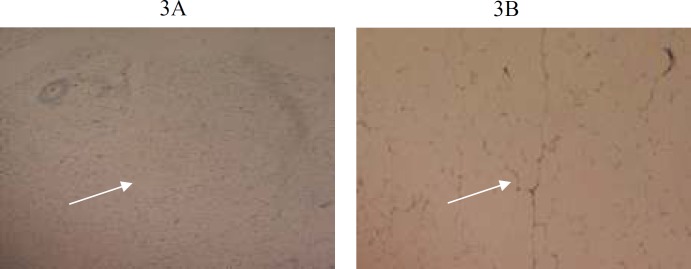
3A, 3B (G1, G3): Photomicrograph of 5 µm section of adipose tissue stained with sudan black staining, showing normal adipocytes at X20 magnification. White arrow indicates adipocytes

G: Grade

**Figure 4:**
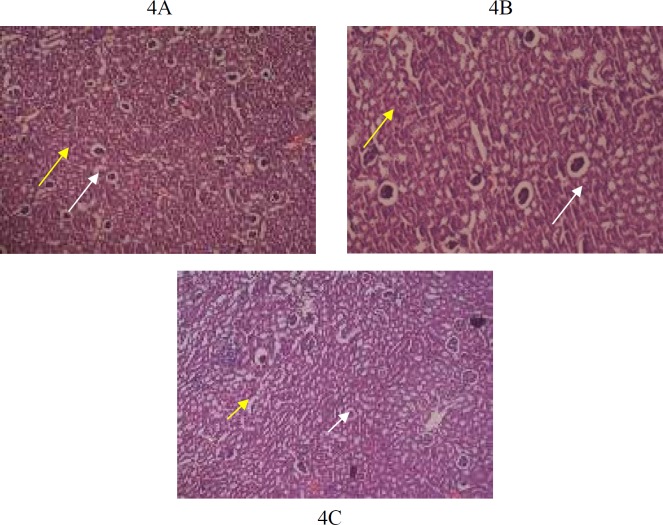
4A (G1): Photomicrograph of 5 µm section of kidney stained with H&E staining, showing normal renal glomeruli (white arrow) and renal tubules (yellow arrow) at X20 magnification

4B: Photomicrograph of 5 µm section of kidney stained with H&E staining, showing normal renal glomeruli (white arrow) and renal tubules (yellow arrow) at X20 magnification

4C: Photomicrograph of 5 µm section of kidney stained with H&E staining, showing hypercellular and atrophic renal glomeruli (white arrow) and dilated renal tubules (yellow arrow) at X20 magnification

G: Grade

**Figure 5:**
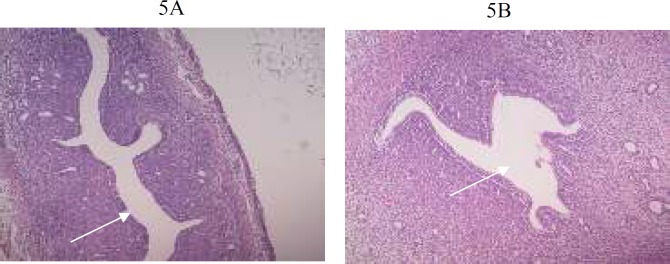
5A, 5B: Photomicrograph of 5 µm section of uterus stained with H&E staining, showing normal uterine cavity (white arrow) at X20 magnification

G: Grade

**Figure 6:**
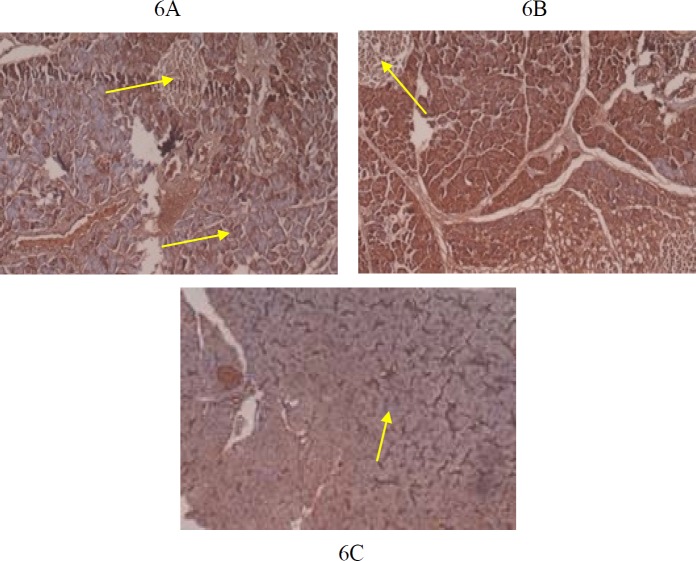
6A, 6B: IHC of Normal pancreatic tissue section showing Ielts of Langerhan’s (Yellow arrow) in G1, G2 stained with anti Ielts 1 (IB1)

6C: IHC of Normal pancreatic tissue section showing shrunkin Ielts of Langerhan’s (Yellow arrow) in G3 stained with anti Ielts 1 (IB1)

G: Grade

**Figure 7:**
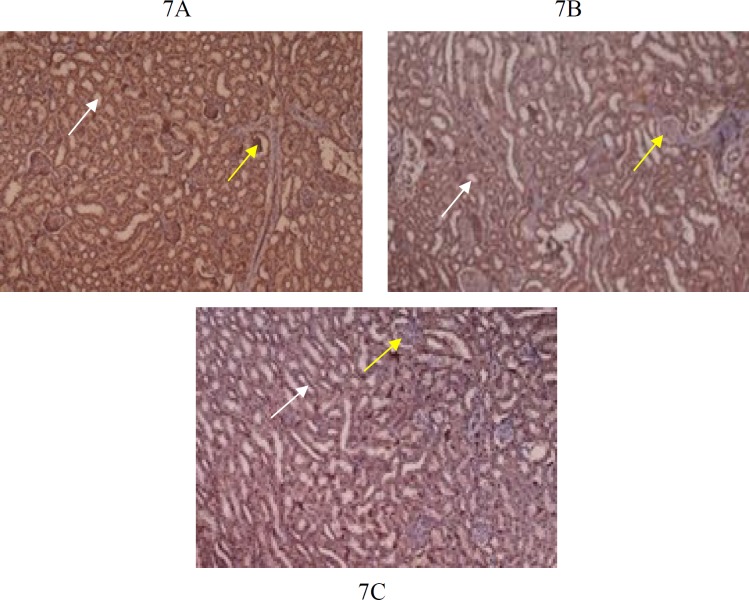
7A, 7B: IHC of G1, G2 shows Normal appearance of kidney with glomeruli (yellow arrow) and tubules (white arrow), stained with KSP- Cadherin

7C: IHC of G3 shows distorted appearance of kidney with hypercellular glomeruli packed with spindle shaped cells (yellow arrow) and atrophic tubules (white arrow), stained with KSP- Cadherin

G: Grade

**Figure 8:**
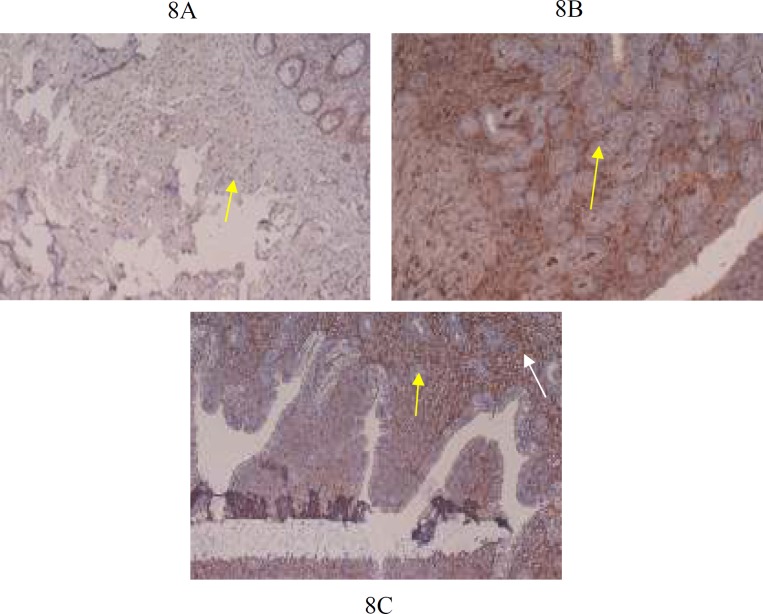
8A (G1): Uterus shows nuclear staining (yellow arrow) with anti MLH- antibody X20 magnification

8B (G1): Uterus shows nuclear staining (yellow arrow) with anti MLH- antibody with x40 magnification

8C (G3): Uterus shows nuclear (yellow arrow) and cytoplasmic staining (white arrow) with anti MLH- antibody X20 magnification

G: Grade

**Table 1:** Grading system for movements of mice

Grade	Criteria
Grade 1	moving quickly around the cage
Grade 2	frequently standing at the sides of cage
Grade 3	active investigation in to surroundings
Grade 4	reduced movement around the cage
Grade 5	little to no investigation around cage
Grade 6	seeks shelter
Grade 7	moves around cage when stimulated
Grade 8	include no movement around cage
Grade 9	may be moribund
Grade 10	typically isolate from cage mates

**Table 2:** Behavior of mice offspring (number=15).

	**G1**	**G2**	**G3**	**X** ^2^ **-value**	***P-*** **value**
1^st^ month	1	2	6	15.5	0.001
2^nd^ month	0	1	5	15.15	0.001
3^rd^ month	0	0	6	14.61	0.003
4^th^ month	0	0	5	16.50	0.0001
5^th^ month	0	0	5	15.15	0.001
6^th^ month	0	0	5	14.61	0.003
7^th^ month	0	0	5	13.50	0.0001
8^th^ month	0	0	5	15.15	0.001
9^th^ month	0	0	4	12.61	0.001
10^th^ month	0	0	4	15.50	0.0001
11^th^ month	0	0	4	14.15	0.001
12^th^ month	0	0	4	13.61	0.0001
13^th^ month	0	0	4	16.50	0.0001
14^th^ month	0	0	4	17.10	0.001
15^th^ month	0	0	3	16.61	0.001

**Table 3:** Movement of mice offspring (number=15) according to criteria mentioned in Table 1

**Time **	**G1**	**G2**	**G3**	**X** ^2^ **-value**	***P-*** **value**
1^st^ month	4	3	10	13.50	0.0001
2^nd^ month	3	3	10	15.15	0.001
3^rd^ month	3	4	9	12.61	0.001
4^th^ month	1	2	8	15.50	0.0001
5^th^ month	2	1	8	14.15	0.001
6^th^ month	1	1	7	13.61	0.0001
7^th^ month	1	1	7	16.50	0.0001
8^th^ month	1	1	6	17.10	0.001
9^th^ month	1	1	6	16.61	0.001
10^th^ month	1	1	5	13.50	0.0001
11^th^ month	1	1	5	15.15	0.001
12^th^ month	1	1	4	12.61	0.001
13^th^ month	1	1	4	15.50	0.0001
14^th^ month	1	1	3	14.15	0.001
15^th^ month	1	1	3	13.61	0.0001

**Table 4:** Body weight and biochemical parameters at 5 month age of all groups

**Parameters**	**Groups**			***P-*** **value**
	**G1**	**G2**	**G3**	
Body weight (g)	8.12±1.11	9.60±0.93	11.80±2.23	0.0001
Glucose (mg/dl)	117.45±13.45	127.08±17.08	171.99±18.99	
Insulin (mIU/l)	9.36±1.27	9.41±1.50	10.92±0.84	
Cholesterol (mg/dl)	118.76±32.25	121.52±3.87	164.90±6.57	
HDLc (mg/dl)	35.17±6.63	34.91±1.67	27.60±1.61	

HDLc: High-density lipoprotein cholesterol.

**Table 5:** Body weight and biochemical parameters at 15 month age of all groups

**Parameters**	**Groups**			***P-*** **value**
	**G1**	**G2**	**G3**	
Body weight (g)	31.54±2.62	31.82±2.96	35.96±11.26	0.0001
Glucose (mg/dl)	116.5±11.5	119.7±18.7	169.8±19.8	
Insulin (mIU/l)	8.92±0.73	9.10±0.84	11.54±1.52	
Cholesterol (mg/dl)	115.02±31.01	120.79±6.51	160.35±7.53	
HDLc (mg/dl)	36.63±6.82	36.0±0.68	28.74±0.52	

HDLc: High-density lipoprotein cholesterol.

***Histological examination***

Histological findings of group G1, G2 and G3 are shown in Figures 1-6**.** Histological examination showed abnormalities in mononuclear lymphocytic infiltration of islets of Langerhansin pancreas (Figure 1C) dilated hypervascular tubules, hypercellularity of glomeruli and few atrophic glomeruli (Figure 4C) in G3. Adipose tissue (Figure 2, 3) and uterine tissue (Figure 5) showed no histological abnormalities in G3.

***Immunohistochemistry***

IHC findings of group G1, G2 and G3 are shown in Figures 6-8. Control groups, G1and G2 showed normal pancreas with anti-islet 1 antibody [1B1] (Figures 6A-6B). Group G3 showed one smaller islet of Langerhans out of 3 visible with anti-islet 1 antibody [1B1](Figure 6C). Normal appearing glomeruli and renal tubules by IHC (Formalin/PFA-fixed paraffin embedded sections) -Ksp-cadherin (Kidney-specific cadherin) were noted in control groups G1and G2 (Figures 7A-7B). Group G3 mice (Ksp-cadherin staining) showed atrophic renal tubules, fibrosed glomeruli and hypercellular glomeruli packed with spindle shaped cells (Figure 7C)**.** Normal nuclear staining of uterine cells with anti-MLH antibody was noted in control groups, G1and G2 (Figures 8A-8B). Abnormal nuclear staining of uterine cells with anti-MLH antibody was noted in group G3. Group G3 showed highly prominent nuclear and cytoplasmic staining with anti-MLH antibody, which indicates nuclear damage (Figure 8C). 

## Discussion

In the present study, the pregnant female mice were exposed to BPA to observe the epigenetic effects in the offspring. Abnormalities of behavior and movement were observed in offspring of experimental group (G3). In present study, the body weight, blood glucose, serum insulin, cholesterol and HDLc were high in group G3 (BPA). High blood glucose in the presence of raised serum levels of insulin indicates endocrine disruption caused by BPA; this type of metabolic abnormality is called the insulin resistance. Many studies (13-16) have reported the prenatal fetal BPA exposure results in altered glucose metabolism during postnatal life. In a similar experiment, the pregnant mice were injected BPA (10 mg/kg/d) on 9 to 16 days of gestation. The offspring revealed glucose intolerance, hyperinsulinemia, insulin resistance and defects in insulin secretion ofpancreatic β-cells compared to control mice at the 6 months of postnatal life (14, 15). At high dose of BPA (5 to 50 mg/kg) during day 9-18^th^ of gestation, the glucose intolerance was noted during postnatal life. These findings corroborated our present research work. Similar findings have been reported by injection of BPA at the dose of 5 and 50 mg/kg/d (16). Postnatal examinationhas also shownthat BPA may decrease insulin sensitivity and lead to hyperinsulinemia (14, 15). 

In present study, the mean blood cholesterol level was high, HDLc was low, and insulin resistance was obvious, indicating an atherogenic milieu in the offspring of BPA-treated female mice. These findings are supported by the results of previous studies (14, 17, 18). Previous studies (17, 18) have reported that the prenatal exposure of BPA with latter high fat diet resulted in severe abnormalities of glucose homeostasis and dyslipidemia similar to what has been noted in the present study i.e. hypercholesterolemia with low HDLc, which are both the risk factor for coronary artery disease. Previous studies (18, 19) have also reported high blood cholesterol, low HDLc, decreased β-cells secretory function and β-cells mass. Ke *et al. *(20) reported that the long term, about 10 months, BPA exposure in male mice resulted in the obesity, dyslipidemia, fatty liver, hypercholesterolemia, and glucose intolerance. Theyfurther worked on the genes of liver lipid metabolism and reported that all these metabolic effects were produced through gene stimulation of key enzymes of fatty acid and cholesterol biosynthesis within the liver resulting in aberrant liver lipid metabolism and dyslipidemia. Ma *et al.* (2013)(21) administered BPA (50 mg/kg/d) torats during pregnancy. During postnatal life, hepatic epigenetic modifications were noted, which resulted in hyperinsulinemia, insulin resistance, impaired glucose tolerance and dyslipidemia. Now,existing evidences indicate that BPA also provokes adverse lipid metabolic consequences in adults (14, 15, 20), which is consistent with our findings in the present research. In the light of above literature review and evidence based findings of the present study, it is suggested that BPA is an endocrine disrupting agent, and its use should be re-evaluated in particular for the food containers. IHC staining with monoclonal antibodies against β-cell islets of Langerhans, kidneys (Ksp-cadherin staining), and uterine anti-MLH antibody proved cell and tissue damage. BPA induced shrinkage of islet of Langerhansand atrophic renal tubules and fibrosed glomeruli. The results of a study (22) on the effects of BPA on fetal pancreasin pregnant mice showed similar histological and functional defects in pancreas of offspring. Pregnant mice were given 25 mg BPA/kg diet from embryonic day. Pancreas of fetal mice were collected and histological examination revealed gross defects in the tissue morphology, such as size and location of islet of Langerhans, and β and α cell distribution. IHC analysis revealed glucagon expression and disturbed islets cell clusters. It was shown that the BPA disturbs the pancreas histology and functions as well (22). Finding of cytoplasmic anti-MLH antibody staining in thepresent research indicated cell injury, which may leadto infertility and neoplastic growth. Li *et al*. (23) studied the effects of chronic exposure of BPA on the uterine functions during early pregnancy in a mice model. They reported that the BPA exposure increased fibroblast growth factor and induced mitogen-activated protein kinase (MAPK) signaling in the endometrium resulting in the aberrant proliferation and uterine receptivity. Differentiation of endometrial cell to deciduas cells was abnormal leading to failure of zygote implantation (23). Findings of the above study are in agreement with present study as the prenatal BPA exposure induced uterine abnormalities, which may adversely affect implantation and the establishment of pregnancy.

The effects of BPA in the containers containing plastic lining food and beverage were studied on the kidney andon the podocytes, and especiallyproteinuria (24). For this purpose, podocytes were exposed to low and high BPA concentrations (10 nM and 100 nM, respectively). Renal histology showed mesangial proliferation. Electron microscopy showed podocytes displacement with an enlargement of foot processes, condensed chromatin, and apoptosis. IHC for WT-1 (specific podocyte marker) by TUNEL technique showed podocytopenia and apoptosis (24). It was concluded the BPA is nephrotoxic and damages the glomerular structure and causes proteinuria. These findings verified the BPA-induced kidney damage, which was presented in the current study. Findings of the present study are in agreement with previous studies, which have reported that the BPA exposure may induce abnormal mammary glands, pre-neoplastic growth, uterine alterations such as the cystic endometrial hyperplasia, cystic ovary, disturbed estrous cycles, prostate gland disease and prostate gland neoplasia (25, 26). BPA affects the meiosis in animals, and interferes with germ cells. BPA is a notorious ovarian toxin, but in present study prenatal exposure of BPA wasshown to damage the uterus as well. This is in agreement with previous study (27), which reported abnormal endometrial proliferation and uterine receptivity. It increases the risk of zygote implantation failure (27). Also, a previous study reported that BPA induces neoplasia and changes in mammary gland tissue (28). The review of above literature confirms the findings of the present study.

## Conclusion

Findings of the current study revealed that the exposure of BPA during intrauterine life alters the epigenetics, which in turn affects the normal development of pancreas, kidneys and uterus in female offspring with resultant progression of adult diseases later in life. 
